# Overexpression of ZNF460 predicts worse survival and promotes metastasis through JAK2/STAT3 signaling pathway in patient with colon cancer

**DOI:** 10.7150/jca.55079

**Published:** 2021-04-02

**Authors:** Tengfei Hao, Jiannan Xu, Sufen Fang, Jianlong Jiang, Xinyuan Chen, Wenhui Wu, Liang Li, Mingzhe Li, Changhua Zhang, Yulong He

**Affiliations:** 1 Digestive Disease Center, The Seventh Affiliated Hospital of Sun Yat-sen University, Shenzhen, Guangdong, 518107, China.; 2 Department of Gastrointestinal Surgery, The First Affiliated Hospital of Sun Yat-sen University, Guangzhou, 510000, China.

**Keywords:** ZNF460, colon cancer, prognostic, metastasis, JAK2/STAT3

## Abstract

Zinc finger proteins (ZNFs) are a class of protein containing zinc finger domains, and they play an important role in tumor progression. However, as a member of the ZNFs family, the effect of ZNF460 in colon cancer remains unclear. In this study, we found that the expression of ZNF460 protein were markedly increased in clinical colon cancer tissues compared with para-cancer non-cancerous tissues by tissue immunohistochemistry (IHC) and western blot (WB). We also confirmed this result at the mRNA and protein levels of ZNF460 through bioinformatics analysis. In addition, high expression of ZNF460 was correlated with increased depth of invasion (P<0.05), increased lymph node metastasis (P<0.05), distant metastasis (P<0.05) and high blood serum CA19-9 level (P<0.05). High expression of ZNF460 predicted poor overall survival (OS) and recurrence free survival (RFS) in patients with colon cancer. Moreover, multivariate analyses revealed that ZNF460 was an independent prognostic factor in both OS (hazard ratio [HR]: 1.636; 95% confidence interval [CI], 1.028-2.603; P = 0.038) and RFS (HR: 2.215; 95% CI: 1.227-3.997; P = 0.008). The knockdown of ZNF460 suppressed the invasion and metastasis of colon cancer cells *in vitro*. Mechanistically, we revealed that ZNF460 promotes the activation of the JAK2/STAT3 signaling pathway in colon cancer cells. Taken together, overexpression of ZNF460 predicted worse survival and promoted metastasis through JAK2/STAT3 signaling pathway in patient with colon cancer, and could be a novel therapeutic target in colon cancer.

## Introduction

Colon cancer is one of the most common types of malignant tumors and ranks fourth in terms of incidence and fifth in terms of mortality worldwide. It is the second most common malignant tumor diagnosed in women and third most in men. The highest colon cancer incidence rates are found in parts of Europe and Australia/New Zealand 1, 2. Currently, surgery is still the most potent curative method for colon cancer 3, 4. But metastasis and recurrence rates after radical resection of colon cancer remained high 5. Furthermore, some colon cancer patients were diagnosed at advanced stages with distant metastasis, which meant missing the window of surgery, thus was associated with shorter survival time and poorer prognosis 6, 7. However, the underlying mechanisms of colon cancer metastasis remain unclear 8. Thus, it is important to investigate the molecular mechanisms involved in colon cancer progression and metastasis.

Zinc finger proteins (ZNFs) are a group of proteins containing zinc finger domains. ZNFs have a wide range of molecular functions and were encoded by 2% of all human genes 9. The Zinc finger structure of protein can bind with gene promoter region and plays an important role in regulating cell proliferation, differentiation, apoptosis and so on 10. ZNFs play an important role in cancer progression or tumor suppression. Previous study has revealed that ZNF306 11, ZNF304 12, and ZEB1 13 contribute to tumorigenesis in colorectal cancer, but ZNF545, as a tumor suppressor, inhibits colon cancer progression 14. ZNF460 as a C2H2-type ZNF, encoding a 562-amino-acid polypeptide, was first identified in 2003 but its function in colon cancer was still unclear 15.

In this study, we first demonstrated that ZNF460 is upregulated in colon cancer tissues compared with para-cancer non-cancerous tissues. High expression of ZNF460 represented poor prognosis and might be associated with metastasis of colon cancer. Multivariate analyses revealed that ZNF460 was an independent prognostic factor in patients with colon cancer. In addition, we also provided the first evidence that ZNF460 promoted the invasion and metastasis of colon cancer cells through activating the JAK2/STAT3 signaling pathway and might be a novel therapeutic target in colon cancer.

## Materials and Methods

### Patients

Surgically treated colon cancer patients (n=262) with confirmed pathology without neoadjuvant chemotherapy at the First Affiliated Hospital of Sun Yat-sen University between 2008 and 2011 were randomly chosen. TNM staging was performed according to the 8^th^ edition of American Joint Committee on Cancer Staging Manual. This study was performed according to the Declaration of Helsinki. Research ethics were approved by the ethical committee of the Seventh Affiliated Hospital of Sun Yat-sen University and the samples were obtained with informed consent.

### Cell culture, siRNAs and reagents

The human colon cancer cell lines SW480, LoVo, DLD-1 and HCT-116 were purchased from the Shanghai Institute of Cell Biology, China. The LS174T cell line was purchased from the ATCC. These cells were cultured in RPMI-1640 medium supplemented with 10% fetal bovine serum (Hyclone, Logan, UT, USA) and antibiotics at 37 °C with 5% CO_2_. Colon cancer cells were transfected with either siZNF460 or empty vector siRNA (Ribo, China) using a riboFETC^TM^ CP transfection reagent (Ribo, China) according to the manufacturer's instructions. The siRNA-transfected colon cancer cells were cultivated at 37 °C for 48 hours. The transfected cells were divided into three experimental groups: Control, NC-siRNA, ZNF460siRNA. After 48 hours, the transfected cells were collected for protein expression analysis. The siRNA sequence is listed as follows: ZNF460siRNA#1: 5′-GCACAGATCTCATTCAAC-3′; ZNF460siRNA#2: 5′-GTCCCAAGATACTCCTATT-3′; ZNF460siRNA#3: 5′- GCCTTCAATTGCCGCTCAT-3′.

### Immunohistochemistry

Paraffin-embedded colon cancer specimens were obtained from the Department of Pathology of First Affiliated Hospital of Sun Yat-sen University. IHC staining were conducted using an anti-ZNF460 antibody (1:200; Sigma-Aldrich, Darmstadt, Germany). Immunoreactivity scores were evaluation by two independent investigators who were blinded to the clinical data of the study and used semiquantitative method to evaluated the scoring. A final agreement between them was obtained for each score, even for discrepant immunostaining results. Histochemistry score (H-SCORE) scoring method was adopted 16. H-SCORE is a scoring method for tissue immunohistochemical results that reflects the positive ratio and the positive intensity. The following formula was used: H-SCORE=∑ (PI × I) = (percentage of cells of weak intensity ×1) + (percentage of cells of moderate intensity ×2) + (percentage of cells of strong intensity ×3). In the formula, PI represents the percentage of positive cells to the total number of cells in this position and I represent the intensity of staining. I=0: blue staining, I=1: light yellow staining, I=2: brown staining and I=3: dark brown staining. The H-SCORE is 0-300, with a higher score representing stronger positive staining.

### Cell migration and invasion assay

For the migration assay, 5×10^4^ LoVo cells and 1×10^5^ LS174T cells were resuspended in serum-free medium and placed in the upper chambers. For the invasion assays, 1×10^5^ LoVo cells and 2×10^5^ LS174T cells were seeded in a Matrigel-coated chamber (BD Biosciences, Bedford, MA, USA). After 36h (to examine migration) or 48h (to examine invasion) of incubation, the non-migrated cells on the upper surface of the membrane were removed, and the cells on the lower surface were fixed and stained with 0.1% crystal violet. The cells in five random microscopic fields were counted and imaged using a light microscope with Leica Microsystems DM4B.

### Bioinformatics analysis

Colon cancer RNA-Seq data and protein expression data were obtained from TCGA data through the UALCAN website (http://ualcan.path.uab.edu) 17. The prognostic role of ZNF460 mRNA levels were analyzed using the TCGA data through the following website (http://bioinfo.henu.edu.cn/DatabaseList.jsp). ZNF460 co-expression was analyzed using Pearson's correlation coefficient, presenting in heatmaps, or scatter plots by using LinkedOmics database 18. Function module of LinkedOmics performed analysis of Gene Ontology biological process (GO_BP) and KEGG pathways with gene set enrichment analysis (GSEA). The rank criterion was FDR < 0.05 and 1,000 simulations were performed.

### Statistical analyses

χ^2^ test, Wilcoxon test and t test was applied for the continuous and discrete data analysis. The associations between ZNF460 expression and overall patient survival were estimated using univariate analysis and the Kaplan-Meier method and further assessed using the log-rank test. Potential prognosis and clinicopathological characteristics were adjusted for using Cox regression models of multivariate analysis, with ZNF460 expression fitted as an indicator variable. All statistical analyses were conducted using the SPSS statistical software (version 22.0; SPSS Inc., Chicago, IL) and GraphPad Prism (version 8.0). All statistical tests were two-sided, and values of p < 0.05 were considered statistically significant.

## Results

### ZNF460 expression is upregulated in colon cancer tissues

**Figure 1:**
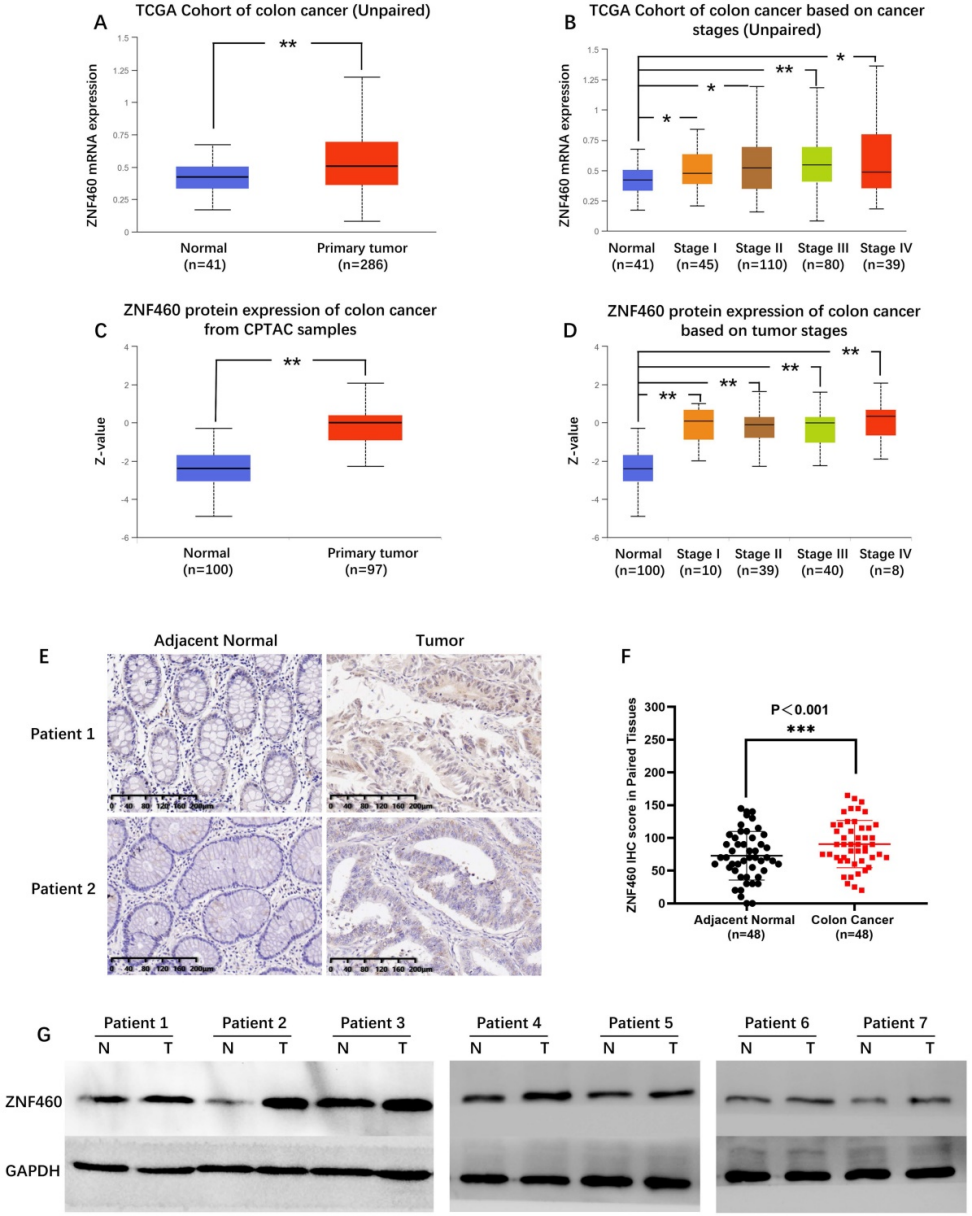
ZNF460 expression is upregulated in colon cancer tissues. (A,B) ZNF460 mRNA expression in unpaired colon cancer and normal tissues from the TCGA database. * P<0.05, ** P<0.01. (C)(D) ZNF460 protein expression in colon cancer and normal tissues from the CPTAC database. ** P<0.01. ZNF460 expression in 48 paired tissues by IHC (E,F) and western blotting (G). *** P<0.001.

Unpaired colon cancer and normal tissues from TCGA indicated ZNF460 mRNA in colon cancer markedly upregulated expression (P<0.001; Fig. 1A). Further analysis of the expression according to tumor stage showed that ZNF460 mRNA were significantly overexpressed in all the stages (P<0.05; Fig. 1B). Consistent with the mRNA expression, data from CPTAC showed that ZNF460 protein expression were also significantly upregulated in colon cancer compared to normal samples (P<0.01; Fig. 1C and Fig. 1D). Further to verify the expression of ZNF460 in colon cancer tissues, we examined ZNF460 expression in 48 paired colon cancer tissue samples and the corresponding adjacent tissues by IHC and WB (1:500; Proteintech, Wuhan, China). The IHC results showed that ZNF460 protein of 79.2% (38/48) patients was highly expressed in the colon cancer tissue samples (Fig. 1E and Fig. 1F). Similarly, WB results also showed the significantly elevated ZNF460 protein levels in the tissues (Fig. 1G), which was consistent with the IHC results. These results indicated that the ZNF460 expression levels were significantly upregulated in colon cancer tissues.

### Relationship between ZNF460 expression and clinical parameters in patient with colon cancer

To further examine the ZNF460 expression and its clinicopathological characteristics in colon cancer, we performed IHC staining in 214 paraffin-embedded colon cancer specimens. The ZNF460 protein was mainly expressed in the cytoplasm and nucleus based on IHC results. The patients were divided into high expression group and low expression group with median (H score = 95) as the cut-off value. The associations of ZNF460 expression with clinical parameters were summarized in Table 1. As shown in the table, ZNF460 expression level was significantly associated with the increased depth of invasion (P<0.05), increased lymph node metastasis (P<0.05), distant metastasis (P<0.05), high CA19-9 level (U/ml) (P<0.05) and TNM stage (P<0.05). These results indicated that the high expression of ZNF460 may be related to the metastasis of colon cancer.

### High expression of ZNF460 predicted worse survival in patients with colon cancer

**Figure 2:**
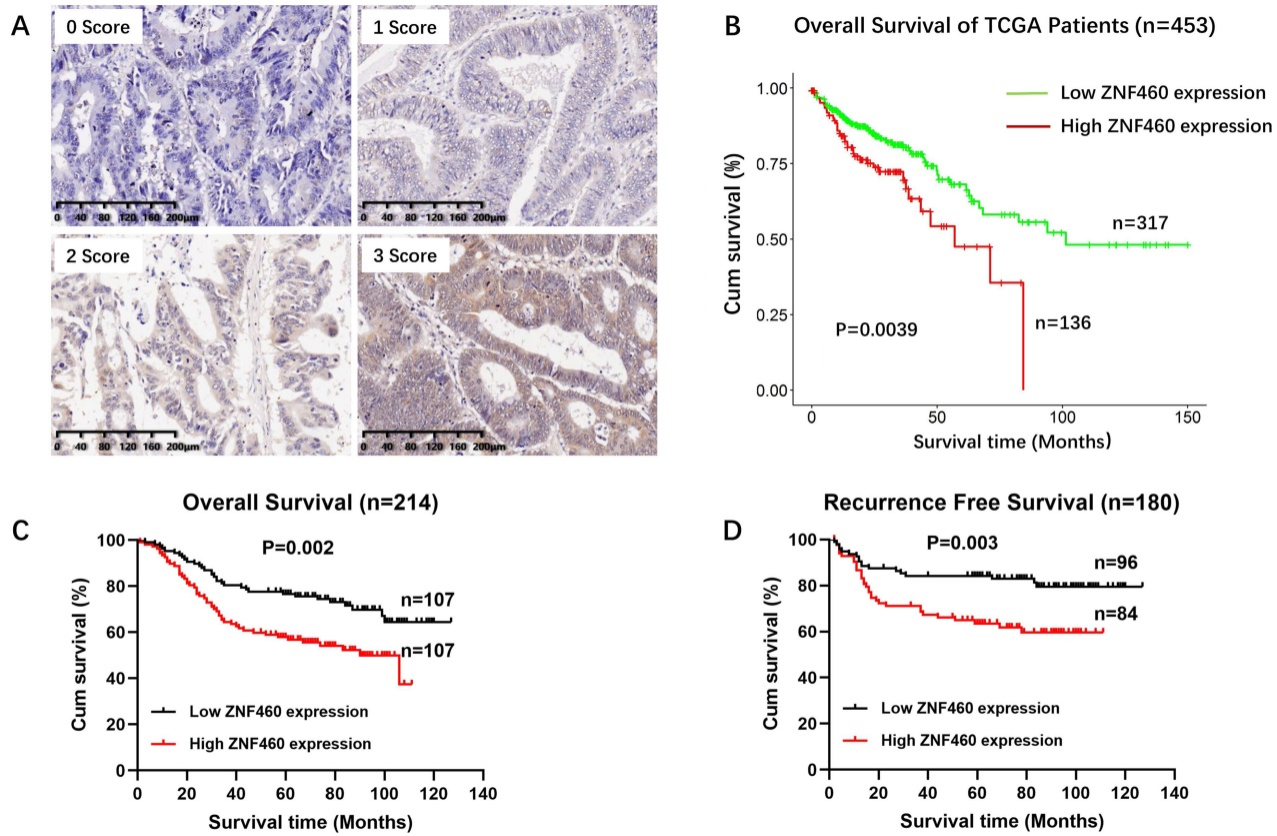
High expression of ZNF460 predicted worse survival in patients with colon cancer. (A) IHC staining, I=0: blue staining, I=1: light yellow staining, I=2: brown staining and I=3: dark brown staining. (B) OS from TCGA database. Log rank P=0.0039. (C) OS from our cohort. Log rank P=0.002. (D) RFS from our cohort. Log rank P=0.003.

To evaluate the prognostic role of ZNF460 expression, we analyzed the prognostic data of ZNF460 using TCGA. As shown in Fig. 2B, the OS were significantly worse in the high ZNF460 group compared to low ZNF460 group (Fig. 2B, log rank P=0.0039). Next, the prognostic value was assessed with Kaplan-Meier analysis of ZNF460 protein expression in our cohort. The follow-up period of the 214 colon cancer patients ranged from 1 to 127 months, with a mean survival time of 65 months. The results outlined that high expression of ZNF460 predicted worse OS (Fig. 2C, log rank P=0.002) and RFS (Fig. 2D, log rank P=0.003) in colon cancer patient. The 5-year OS rate was 76.6% in the low ZNF460 expression group and 57.9% in the high ZNF460 expression group, consistent with the TCGA data. The mean survival times of patients with low and high ZNF460 expression were 74 and 57 months, respectively.

We also performed univariate and multivariate analyses of various clinical data to assess the prognostic factor. As shown in Tables 2 & 3, there are various parameters associated with OS and RFS in colon cancer. Further Cox proportional-hazards regression analysis showed that ZNF460 expression was an independent prognostic factor for OS (Table 2; HR=1.636, 95% CI: 1.028-2.603; P = 0.038) and RFS (Table 3; HR: 2.215; 95% CI: 1.227-3.997; P = 0.008). Taken together, the data indicated that ZNF460 high expression predicted a worse survival and was an independent prognostic factor for the patients with colon cancer.

### High expression of ZNF460 might be associated with lymph node metastasis of colon cancer

**Figure 3:**
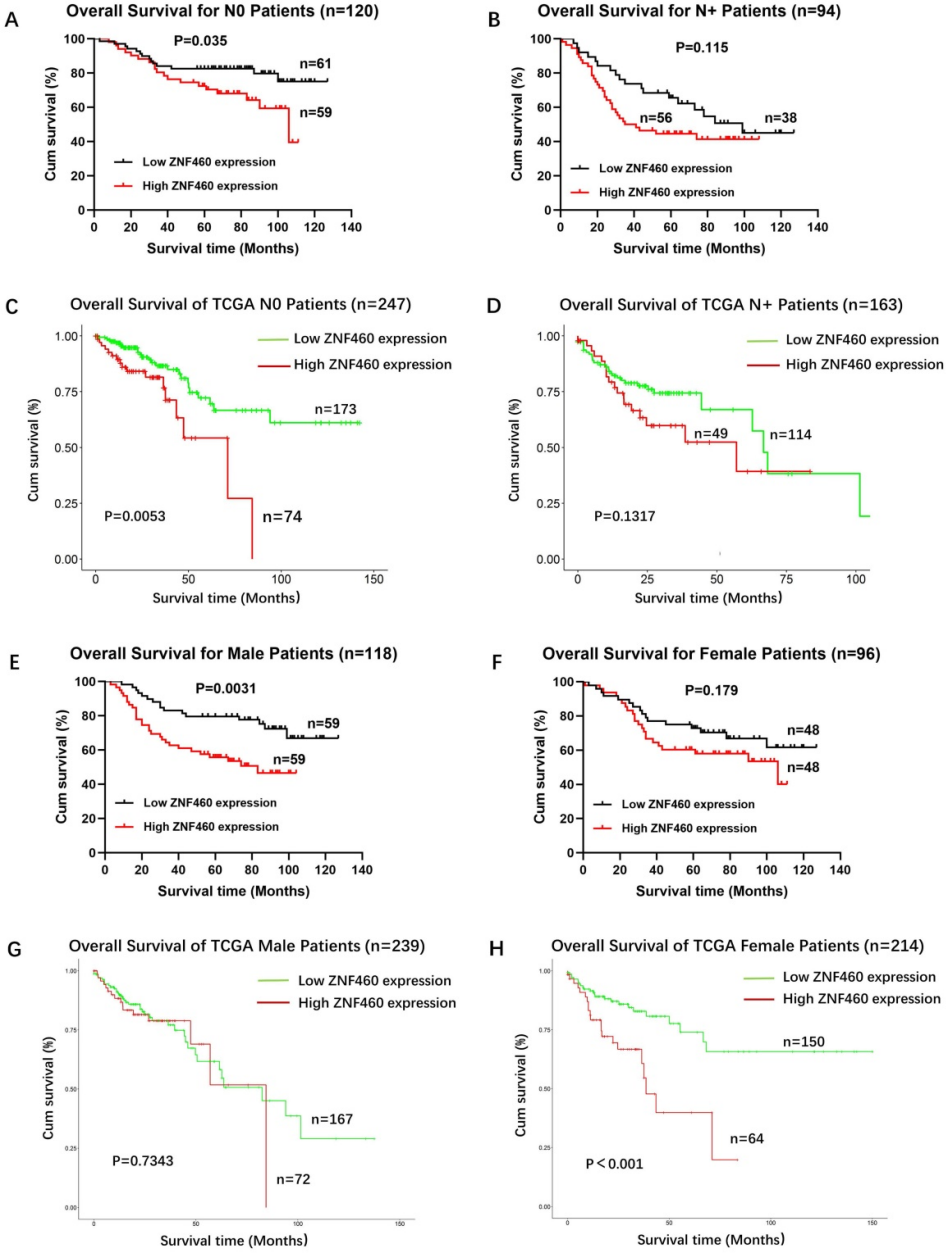
High expression of ZNF460 may be associated with lymph node metastasis of colon cancer. (A) OS for N0 patients. Log rank P=0.035. (B) OS for N+ patients. Log rank P=0.115. (C) OS for N0 patients from TCGA database. Log rank P=0.0053. (D) OS for N+ patients from TCGA database. Log rank P=0.1317. (E) OS for male patients. Log rank P=0.0031. (F) OS for female patients. Log rank P=0.179. (G) OS for male patients from TCGA database. Log rank P=0.7343. (H) OS for female patients from TCGA database. Log rank P<0.001.

We defined the prognostic value of ZNF460 expression in N0 stage and N+ (N1, N2) stages. The results showed that high expression of ZNF460 associated with worse OS (Fig. 3A, log rank P = 0.035) in N0 stage, but it had no statistical difference in N+ stages (Fig. 3B, log rank P = 0.115). These are consistent with the analysis results of TCGA database (N0: Fig. 3C, log rank P=0.0053; N+: Fig. 3D, log rank P=0.1317). We also found that high ZNF460 expression associated with worse OS in male patients (Fig. 3E, log rank P = 0.0031), but had no statistical significance in female patients (Fig. 3F, log rank P = 0.179). However, based on TCGA analysis, high ZNF460 expression was associated with worse OS in female instead (Fig. 3H, log rank P < 0.001) but not male patients (Fig. 3G, log rank P=0.7343). In conclusion, these results suggested that overexpression of ZNF460 was not only associated with lymph node metastasis of colon cancer, but also with geographical region and race.

### Downregulation of ZNF460 represses migration and invasion in colon cancer cells

**Figure 4:**
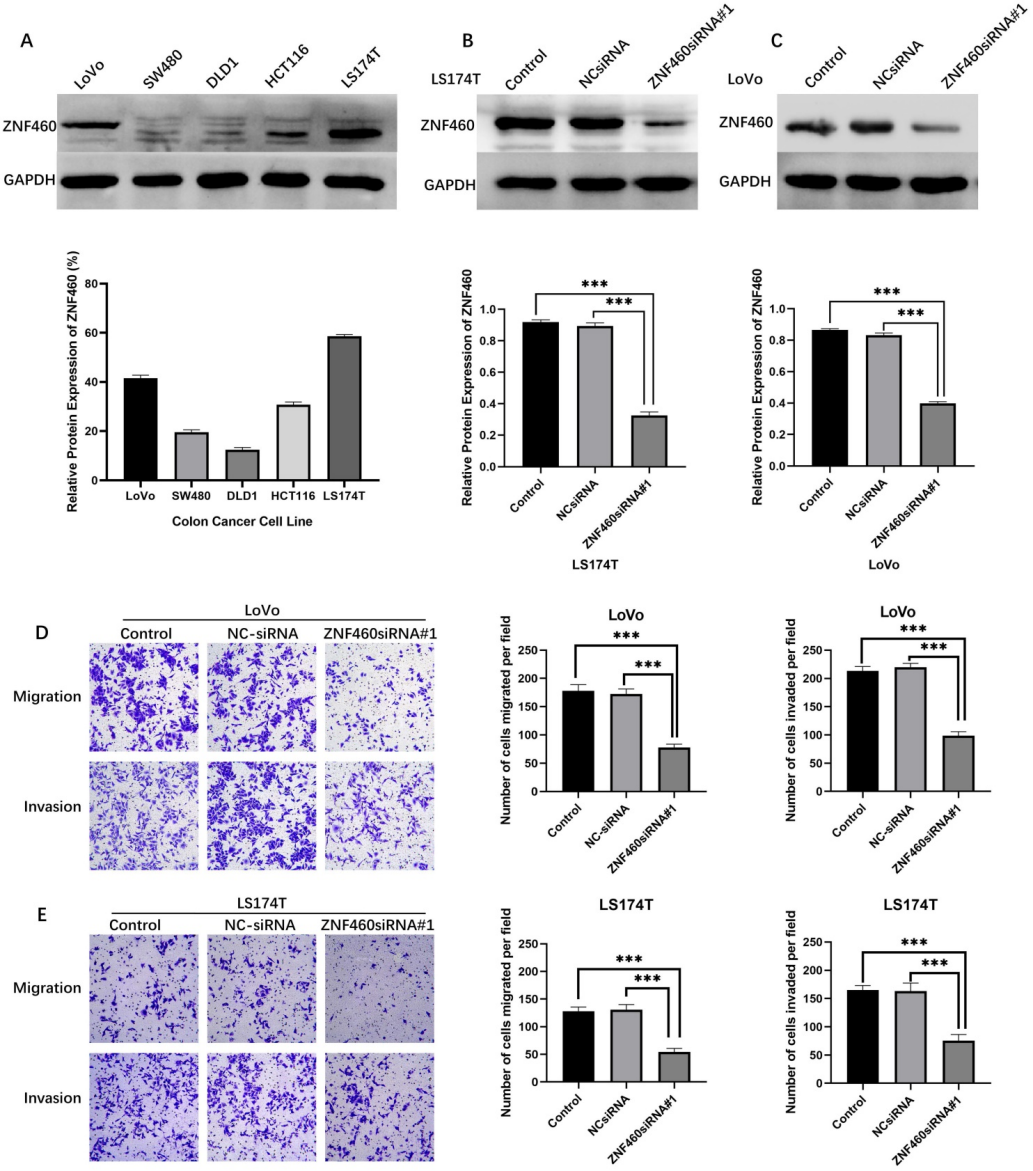
Downregulation of ZNF460 represses migration and invasion in colon cancer cells. (A) Expression level of ZNF460 in colon cancer cells. (B,C) LS174T and LoVo transfected with ZNF460siRNA#1. *** P<0.001. (D) Alteration of LoVo migration and invasion ability. *** P<0.001. (E) Alteration of LS174T migration and invasion ability. ***P<0.001.

To further investigate the functions of ZNF460 in colon cancer cell invasion and migration, we first use WB analyses of ZNF460 expression in the indicated colon cancer cell lines. The results exhibited relatively high expression of ZNF460 among LS174T and LoVo cells (Fig. 4A). Then we manipulated the ZNF460 levels by transfecting ZNF460 siRNA and empty vector siRNA into LS174T (Fig. 4B) and LoVo (Fig. 4C) cells. Finally, we found that ZNF460-siRNA#1 had the best knockdown effect, which were shown in Fig. 4B and 4C. We found that cells with knockdown of ZNF460 displayed a significant decrease in cell migration and invasion abilities compared to cells transfected with the vector control (Fig. 4D).

### ZNF460 co-expression networks and GO/KEGG biological process enrichment

**Figure 5:**
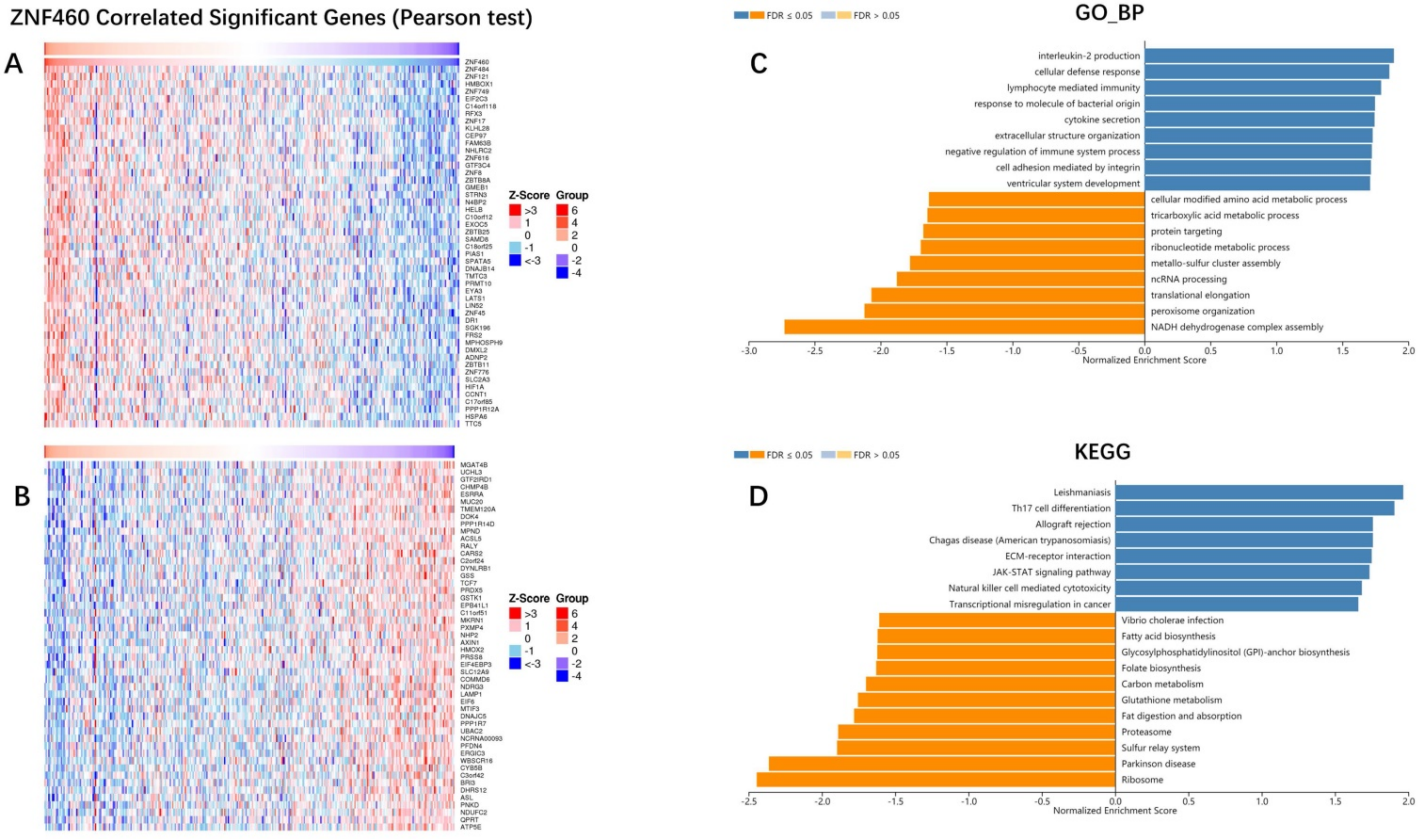
ZNF460 co-expression genes in colon cancer (LinkedOmics). (A,B) Heatmaps showing top 50 genes positively and negatively correlated with ZNF460 in colon cancer cohort. Red indicates positively correlated genes and blue indicates negatively correlated genes. (C,D) Significantly enriched GO annotations and KEGG pathways of ZNF460 in colon cancer cohort.

To further explore the mechanism of ZNF460 in promoting the invasion and metastasis of colon cancer, LinkedOmics was used to examine ZNF460 co-expression genes in colon cancer cohort. The top 50 significant genes positively and negatively correlated with ZNF460 were shown in the heatmap (Figure 5A, B). The gene with the strongest positive correlation with the expression of ZNF460 was ZNF484, and the strongest negative correlation was MGAT4B.

Significant Gene Ontology (GO) term annotation by gene set enrichment analysis 17 (GSEA) showed that ZNF460 co-expressed genes participate primarily in interleukin-2 production, cellular defense response, and lymphocyte mediated immunity, while the NADH dehydrogenase complex assembly, and peroxisome organization were inhibited (Figure 5C). Kyoto Encyclopedia of Genes and Genomes (KEGG) pathway analysis showed enrichment in the ECM-receptor, and JAK-STAT signaling pathway, etc. (Figure 5D). These results showed that ZNF460 played an extensive role in the progression of colon cancer.

### ZNF460 promotes metastasis through JAK2/STAT3 signaling pathway

**Figure 6:**
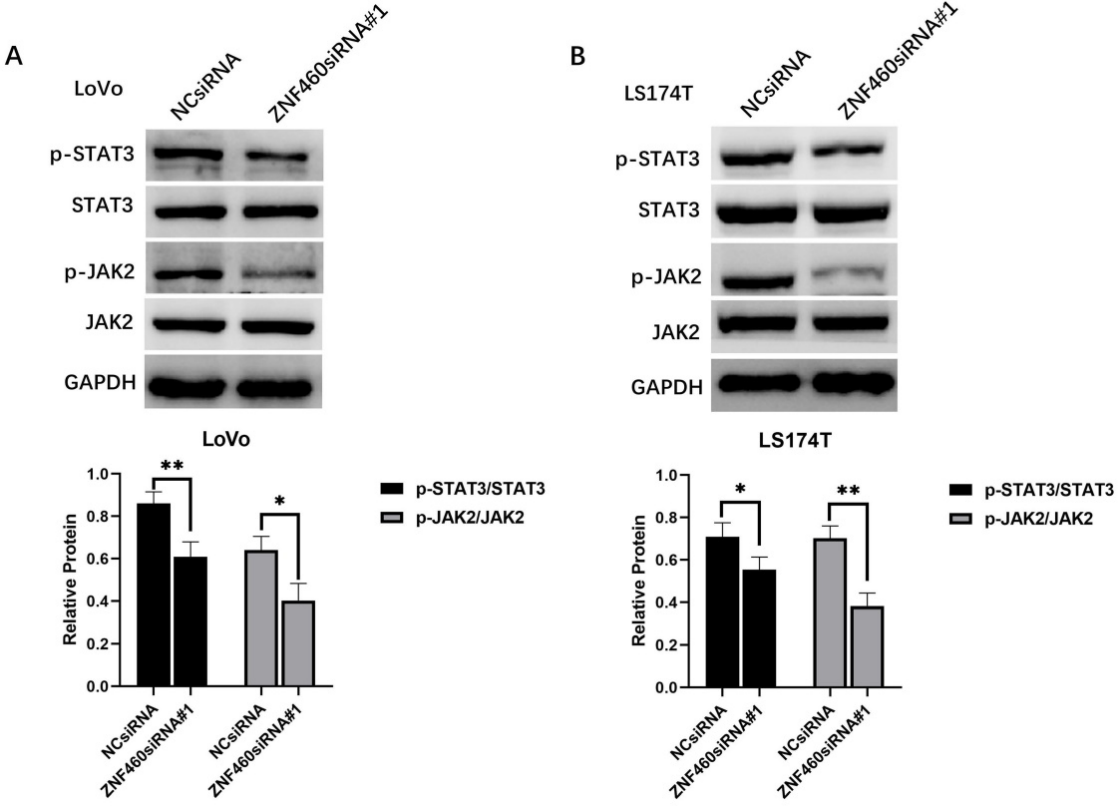
ZNF460 promotes metastasis through JAK2/STAT3 signaling pathway. (A) Expression level of p-STAT3/STAT3 and p-JAK2/JAK2 in LoVo. * P<0.05, ** P<0.01. (B) Expression level of p-STAT3/STAT3 and p-JAK2/JAK2 in LS174T. * P<0.05, ** P<0.01.

According to the results of bioinformatics analysis, ZNF460 may promote the metastasis of colon cancer by activating the JAK/STAT signaling pathway. To further elucidate the mechanism, western blot was utilized to detect protein expression of p-JAK2, JAK2, p-STAT3 and STAT3 in colon cancer cells after transfection with ZNF460 siRNA. The results showed that downregulation of ZNF460 expression significantly decreased the p-JAK2 and p-STAT3 expression levels in colon cancer cells (Figure 6). This result showed that ZNF460 promotes metastasis through JAK2/STAT3 signaling pathway.

## Discussion

ZNF460 contains a KRAB A+B box and eleven C2H2 type zinc finger motifs which was first described in 2003 15. Previous studies had demonstrated that ZNFs could recruit different chromatin modifiers and served diverse regulation mechanisms. For example, ZNFs could recruit co-repressors and play a role of transcriptional repressor 19. On the other hand, it could work as transcriptional activators by interacting with co-activators, including CBP/p300 and C/EBP 20. However, the expression of ZNF460 in colon cancer and its function in the process of colon cancer invasion and metastasis remained unclear. In this study, we first showed that ZNF460 was overexpressed in colon cancer specimens, was significantly correlated with poor survival, and promoted the invasion and metastasis of colon cancer. Multivariate Cox regression analysis demonstrated ZNF460 expression to be an independent prognostic factor for OS and RFS in colon cancer patients. Mechanistically, we revealed that ZNF460 promotes metastasis through JAK2/STAT3 signaling pathway in colon cancer cells. These results confirmed that ZNF460 was a potential prognostic biomarker and therapeutic target in patients with colon cancer.

Previous studies had reported that several ZNFs were associated with tumor prognosis. To be exact, the zinc finger and BTB domain-containing 7C (ZBTB7C) had been reported to be downregulated in colorectal cancer and could act as an independent prognostic factor 21. The Zinc-fingers and homeoboxes 3 (ZHX3) and ZNF93 had been reported to be significantly increased and were associated with poor prognosis in gastric cancer 22 and ovarian cancer 23, respectively. Consistent with results of previous studies, our study showed for the first time that ZNF460 expression was significantly upregulated in colon cancer tissues at both the mRNA and protein levels, which was associated with poor prognosis and could be an independent prognostic factor in colon cancer patients. We also found that high expression of ZNF460 had a worse prognosis in male than female. This result was contradictory to the TCGA result, suggesting that it might be related to geography and race.

In recent years, more and more ZNFs were identified as oncogene and promoted various tumors via enhancing cell proliferation 24, promoting cell migration and invasion 25, suppressing autophagy 26, and epithelial-mesenchymal transition 27. ZNF460 gene co-expression network analysis established that ZNF460 had a positive correlation with ZNF484, ZNF121, HMBOX1 and a negative correlation with MGAT4B, UCHL3, GTF2IRD1. It had been confirmed that ZNF121 and HMBOX1 played a role in promoting cancer proliferation in breast cancer and gastric cancer 28, 29. Therefore, we speculated that ZNF121 and HMBOX1 played a collaborative role with ZNF460 in carcinogenesis of colon cancer.

Many studies had confirmed that ZNFs family promoted the progression of colorectal cancer. Xing et al. found that ZNF692 promoted colon adenocarcinoma cell growth and metastasis by activating the PI3K/AKT pathway 30, and Qin et al. also found that ZNF281 regulates cell proliferation, migration and invasion in colorectal cancer through Wnt/β-Catenin signaling 31. Consistent with previous studies, our study also indicated ZNF460 promoted colon cancer invasion and migration, especially lymph node metastasis. To further explore the mechanism of ZNF460 in promoting the invasion and metastasis of colon cancer, we found that ZNF460 activated JAK2/STAT3 signaling pathway. These results suggested that ZNF460 promoted invasion and metastasis by activating the JAK-STAT signaling pathway. Similar to our results, Daniel Triner et al. reported that myc-associated zinc finger (MAZ) protein regulated the proinflammatory response in colon cancer via STAT3 signaling 32, and Vikas Verma et al. reported that the activity of JAK-STAT signaling pathway was affected by artificially altering the expression level of ZNF143 33. Zeng YT et al. also found that ZFP42 zinc finger protein promoted metastasis of cervical cancer by upregulating the activity of the JAK2/STAT3 pathway 34.

In summary, we found that ZNF460 was overexpressed in colon cancer, and its expression was correlated with clinical outcome. ZNF460 overexpression was significantly associated with poor prognosis and increased metastatic capabilities of human colon cancer by activating JAK2/STAT3 signaling pathway, and ZNF460 might act as a potential prognostic biomarker and therapeutic target.

## Figures and Tables

**Table 1:** Associations of ZNF460 expression with clinical parameters in 214 colon cancer patients

Characteristic	No.	ZNF460 expression		P value
		Low (N=107)	High (N=107)	
**Age (year)**		58.0±14.0	59.3±12.3	0.584
≤60y	101	53	48	
>60y	113	54	59	
**Gender**				1.000
Male	118	59	59	
Female	96	48	48	
**Tumor location**				0.681
Right hemicolon	98	47	51	
Left hemicolon	116	60	56	
**Differentiation**				0.738
Well+ Moderate	169	83	86	
Poor	45	24	21	
**Depth of invasion**				0.006
T1	2	1	1	
T2	20	15	5	
T3	116	61	55	
T4	76	30	46	
**Lymph node metastasis**				0.025
N0	120	69	51	
N1	65	25	40	
N2	29	13	16	
**Distant metastasis**				0.039
M0	180	96	84	
M1	34	11	23	
**TNM stage**				0.001
I	22	16	6	
II	84	48	36	
III	74	32	42	
IV	34	11	23	
**CEA level (μg/L)**				0.126
≤5	126	69	57	
>5	88	38	50	
**CA19-9 level (U/ml)**				0.048
≤35	176	94	82	
>35	38	13	25	
**Vessel or nerve invasion**				0.147
Yes	37	14	23	
No	177	93	84	
**Chemotherapy or not**				0.055
Yes	109	47	62	
No	105	60	45	

**Table 2:** Cox proportional-hazard regression analysis for Overall Survival in 214 patients with colon cancer

Characteristic	Univariate analysis				Multivariate analysis			
	*P*-value	HR	95.0% CI for Exp (B)		*P*-value	HR	95.0% CI for Exp (B)	
			Lower	Upper			Lower	Upper
**Gender**	0.971	1.008	0.654	1.553				
Age	0.911	0.976	0.634	1.502				
Tumor location	0.243	0.774	0.503	1.190				
Differentiation	0.000	2.286	1.441	3.625	0.038	1.739	1.032	2.931
**Depth of invasion**	0.014	5.775	1.420	23.496				
T1+T2								
T3+T4								
**Lymph node metastasis**	0.000	2.486	1.600	3.865				
N0								
N+								
Distant metastasis	0.000	6.176	3.843	9.925				
**TNM stage**	0.000	3.624	2.236	5.874	0.000	2.979	1.719	5.163
I+II								
III+IV								
CEA level	0.017	1.691	1.099	2.601	0.201	1.350	0.853	2.137
CA19-9 level	0.100	1.550	0.919	2.615				
Vessel or nerve invasion	0.000	5.493	3.461	8.716	0.001	2.610	1.512	4.506
Chemotherapy or not	0.681	1.095	0.711	1.687	0.003	2.013	1.264	3.208
**ZNF460 expression**	0.003	1.967	1.261	3.069	0.038	1.636	1.028	2.603
Low								
High								

**Table 3:** Cox proportional-hazard regression analysis for Recurrence Free Survival in180 patients with colon cancer

Characteristic	Univariate analysis				Multivariate analysis			
	*P*-value	HR	95.0% CI for Exp (B)		*P*-value	HR	95.0% CI for Exp (B)	
			Lower	Upper			Lower	Upper
Gender	0.186	1.458	0.834	2.550				
Age	0.720	0.903	0.519	1.574				
Tumor location	0.902	0.966	0.552	1.688				
Differentiation	0.009	2.234	1.219	4.095	0.083	1.892	0.921	3.889
**Depth of invasion**	0.039	8.019	1.107	58.078				
T1+T2								
T3+T4								
**Lymph node metastasis**	0.007	2.163	1.236	3.785				
N0								
N+								
**TNM stage**	0.007	2.163	1.236	3.785	0.109	1.654	0.893	3.061
I+II								
III								
CEA level	0.025	1.885	1.083	3.284	0.040	1.807	1.029	3.175
CA19-9 level	0.679	1.173	0.551	2.500				
Vessel or nerve invasion	0.001	3.613	1.689	7.726	0.268	1.671	0.673	4.149
Chemotherapy or not	0.288	1.354	0.774	2.368	0.662	1.143	0.627	2.085
**ZNF460 expression**	0.004	2.321	1.300	4.141	0.008	2.215	1.227	3.997
Low								
High								
